# Light-emitting diodes: brighter NIR-emitting phosphor making light sources smarter

**DOI:** 10.1038/s41377-020-00394-5

**Published:** 2020-09-03

**Authors:** Rong-Jun Xie

**Affiliations:** College of Materials, Xiamen University, Xiamen, 361005 Fujian China

**Keywords:** Inorganic LEDs, Optical materials and structures

## Abstract

A brighter near-infrared (NIR) phosphor is achieved by inhibiting the oxidation of Cr^3+^ and reducing the surface defects of phosphor particles, enabling the realization of smarter and more sensitive light sources for night vision.

Near-infrared (NIR) spectroscopy is an emerging and powerful technology for studying organic matter, such as food and biological tissues. It can be used to quickly and nondestructively detect organic components by taking advantage of the characteristic absorption signals of C–H, O–H, and N–H in the spectral range of 700–1100 nm^1,2^. This technology plays key roles in quality monitoring for foods and medicines, bioimaging, and night vision. Very recently, small NIR light sources have been proposed to be applied in smart phones to enable the fast and convenient detection of the freshness and safety of food such as meat, fruits, and vegetables^3^. Among traditional light sources such as tungsten filament lamps and halogen lamps, only light-emitting diodes (LEDs) are suitable for use in smart NIR devices because of their solid-state and compact nature. However, NIR-LED chips usually emit quite a narrow band of NIR light, which hinders their sensitivity and breadth of application^4^. To overcome this disadvantage, an alternative solution is to combine broadband NIR phosphor(s) with a blue LED chip, yielding a device known as an NIR-phosphor-converted (pc) LED. Therefore, the search for and development of highly efficient NIR phosphors that can be excited by blue light represent an important challenge^5^.

The transition metal Cr^3+^ is an ideal NIR emitter, and several Cr^3+^-activated NIR phosphors have been developed for smart LEDs^1,5–9^. Among these, garnet-type phosphors have been intensively investigated due to their unique capability of luminescence regulation^5–9^. However, the overall performance of the garnet NIR phosphors reported so far is still not satisfactory for particle applications. For example, for monitoring and detection, NIR phosphors should efficiently emit light in an appropriate spectral range to guarantee good sensitivity (i.e., a high conversion efficiency) and show low-temperature sensitivity in their luminescence to ensure device reliability (i.e., low thermal quenching). Several strategies, including energy transfer and cationic substitution, have been proposed that can greatly enhance the conversion efficiency or enable the manipulation of spectral position as well as bandwidth, thereby permitting the realization of NIR-LEDs with high radiant power^6–8^. However, when there is no need to modify the emission position of a phosphor, these methods are not the first choice. Liu and colleagues have developed a facile way to significantly improve the quantum efficiency and thermal stability of Cr^3+^-doped silicate garnet Ca_3_Sc_2_Si_3_O_12_ (CSSG:Cr^3+^) while maintaining its spectral shape and position^10^.

Differing from previous approaches, the authors make it easier to control the trivalent state of Cr, as well as the crystallinity and morphology of the phosphor particles, by firing the sample in a CO reducing atmosphere to prevent the oxidation of Cr^3+^ and using an appropriate additive to obtain defect-free phosphors. As a result, the prepared CSSG:Cr^3+^ exhibits an internal quantum efficiency of up to 92.3% and maintains its luminescence efficiency of 97.4%, even at 150 °C. When this CSSG:Cr^3+^ is combined with a high-power 460 nm blue chip, the resulting pc-NIR-LED exhibits a world-record radiant power of 109.9 mW at a driving current of 520 mA, making this excellent pc-NIR-LED suitable for use in night vision applications.

As Cr^3+^ is an important and commonly used activator for NIR phosphors, regulating the luminescence of Cr^3+^ and making practical use of it remain critical challenges to address. The strategy proposed by Liu et al. offers impactful insights for the preparation of highly efficient Cr^3+^-doped NIR phosphors and high-sensitivity smart NIR-LEDs.
